# Supplementary material for: Neural activity related to volitional regulation of cortical excitability
Source: eLife. 2018 Nov 29;7:e40843. doi: 10.7554/eLife.40843 (PMC6294548; doi:10.7554/eLife.40843)
Supplement: Supplementary file 1. — Also shown are accuracies of ‘null models’ with random label permutation performed on the same data, along with statistical comparison across subjects of true accuracies vs randomly permuted accuracies (Wilcoxon signed rank tests). Supplementary Table 2: F tests following mixed effects models comparing background EMG across conditions and blocks. Shown is feedback type (UP,DOWN) by block number interactions on each session. Supplementary Table 3: Percentage of EMG trials retained. Shown is the percentage (of total number of collected MEP trials) that were retained per block and per session for analysis following post-processing screening of background EMG amplitude. Supplementary Table 4: Results of mixed effects models on the percentage of retained background EMG trials during neurofeedback. The dependant variable was the percentage of trials that were retained following screening for background EMG crossing the criterion threshold. Four models were performed; One on data during all neurofeedback trials (10 levels of ‘trial’) modelling changes over the duration of training (across trials) that were modulated by trial type (UP or DOWN), demonstrating no systematic effects of MEP amplitude modulation on the quantity of trials that were retained for analysis. The three remaining models were conducted separately on the data from Session 1, Session two and Session 3, again revealing no significant modulation on any session. Mixed models employed a compound symmetry covariance matrix, fixed effects of ‘trial’ and ‘trial type’, and random effect of ‘participant’. Supplementary Table 5: F tests following mixed effects models comparing background EMG across conditions and blocks for the retention test conducted months following initial training. Shown is feedback type (UP, DOWN) by block number two levels, baseline + retention) interactions for each of the four recorded muscles. Supplementary Table 6: In debriefing following the experiment, all participants were asked to wr [file elife-40843-supp1.docx]

Supplementary Material

| **Classification model inputs** | **Mean Accuracy** | **SD Accuracy** | **Random Permutation Accuracy** | **True Acc vs Random Acc** |
| --- | --- | --- | --- | --- |
| All 8 hotspot rhythms + all opp hotspot +ratios | 85.10% | 4.60% | 52.40% | p<0.001 |
| All 8 hotspot rhythms + all opp hotspot | 85.06% | 3.84% | 49.70% | p<0.001 |
| All 8 hotspot rhythms plus ratio | 81.50% | 5.10% | 49.60% | p<0.001 |
| All 8 opp hotspot rhythms | 79.08% | 4.41% | 50.40% | p<0.001 |
| All 8 hotspot rhythms | 78.89% | 1.69% | 49.00% | p<0.001 |
|  | | | | |
| **Top model: All 8 hotspot rhythms + all opp hotspot +ratios** | | | | |
| **Feature Rank** | **EEG Feature** | | | |
| 1st | H gamma hotspot' | | | |
| 2nd | H alpha hotspot' | | | |
| 3rd | LowGamma:HighAlpha ratio hotspot' | | | |
| 4th | L gamma hotspot' | | | |
| 5th | L gamma hotspot' | | | |
| 6th | Delta opphotspot' | | | |
| 7th | L alpha opphotspot' | | | |
| 8th | delta opphotspot' | | | |
| 9th | L gamma opphotspot' | | | |
| 10th | L alpha opphotspot' | | | |
| 11th | theta opphotspot' | | | |
| 12th | delta hotspot' | | | |
| 13th | delta hotspot' | | | |
| 14th | theta opphotspot' | | | |
| 15th | theta opphotspot' | | | |
| 16th | theta hotspot' | | | |
| 17th | H beta hotspot' | | | |
| 18th | theta opphotspot' | | | |

**Supplementary Table 1**.

| **Experimental Group** | | | | | |
| --- | --- | --- | --- | --- | --- |
| **FDI (target muscle)** | | | | | |
| **Session** | **F** | **df (num)** | **df (denom)** | **p** | **n** |
| **Training Ses1** | 1.28 | 4.00 | 109.7 | 0.279 | 15 |
| **Training Ses2** | 0.95 | 4.00 | 130.5 | 0.439 | 15 |
| **EEG Session** | 1.77 | 2 | 90.93 | 0.175 | 15 |
|  | | | | | |
| **Control Group** | | | | | |
| **FDI (target muscle)** | | | | | |
| **Session** | **F** | **df (num)** | **df (denom)** | **p** | **n** |
| **Training Ses1** | 0.974 | 4.00 | 108 | 0.425 | 13 |
| **Training Ses2** | 1.58 | 4.00 | 108 | 0.182 | 13 |
| **EEG Session** | 2.57 | 2 | 60 | 0.085 | 13 |
|  | | | | | |
| **Control muscles (Experimental group)** | | | | | |
| **OP** | | | | | |
| **Session** | **F** | **df (num)** | **df (denom)** | **p** | **n** |
| **Training Ses1** | 0.422 | 4.00 | 110.87 | 0.792 | 15 |
| **Training Ses2** | 1.91 | 4.00 | 120.985 | 0.112 | 15 |
| **EEG Session** | 0.203 | 2 | 77.06 | 0.817 | 15 |
|  | | | | | |
|  | | | | | |
| **ADM** |  | | | | |
| **Session** | **F** | **df (num)** | **df (denom)** | **p** | **n** |
| **Training Ses1** | 0.526 | 4 | 111.006 | 0.716 | 15 |
| **Training Ses2** | 0.533 | 4 | 123.057 | 0.712 | 15 |
| **EEG Session** | 0.163 | 2 | 70 | 0.85 | 15 |
|  | | | | | |
| **Left FDI** |  |  |  |  |  |
| **Session** | **F** | **df (num)** | **df (denom)** | **p** | **n** |
| **Training Ses1** | 0.361 | 4 | 111.017 | 0.836 | 15 |
| **Training Ses2** | 0.29 | 4 | 125.006 | 0.884 | 15 |
| **EEG Session** | 0.872 | 2 | 70 | 0.423 | 15 |

**Supplementary Table 2: F tests following mixed effects models comparing background EMG across conditions and blocks**

|  | Session1 | | | | Session 2 | | | | EEG session | |
| --- | --- | --- | --- | --- | --- | --- | --- | --- | --- | --- |
| Overall | Trial 1 | Trial 2 | Trial 3 | Trial 4 | Trial 5 | Trial 6 | Trial 7 | Trial 8 | Trial 9 | Trial 10 |
| mean | 70.25 | 72.18 | 66.41 | 67.82 | 70.80 | 69.22 | 72.11 | 71.56 | 72.67 | 73.78 |
| SEM | 4.98 | 5.02 | 4.92 | 4.72 | 4.73 | 4.80 | 4.12 | 4.41 | 4.77 | 4.17 |
|  | | | | | | | | | | |
|  | Session1 | | | | Session 2 | | | | EEG session | |
| UP blocks | Trial 1 | Trial 2 | Trial 3 | Trial 4 | Trial 5 | Trial 6 | Trial 7 | Trial 8 | Trial 9 | Trial 10 |
| mean | 69.23 | 68.97 | 59.49 | 60.51 | 72.44 | 68.22 | 68.67 | 70.89 | 65.78 | 69.56 |
| SEM | 5.89 | 6.36 | 6.18 | 5.76 | 6.70 | 6.81 | 5.25 | 6.45 | 7.03 | 6.41 |
|  | | | | | | | | | | |
|  | Session1 | | | | Session 2 | | | | EEG session | |
| DOWN blocks | Trial 1 | Trial 2 | Trial 3 | Trial 4 | Trial 5 | Trial 6 | Trial 7 | Trial 8 | Trial 9 | Trial 10 |
| mean | 71.19 | 75.38 | 73.33 | 75.13 | 69.05 | 70.22 | 75.56 | 72.22 | 79.56 | 78.00 |
| SEM | 8.11 | 7.93 | 7.40 | 7.12 | 6.89 | 7.00 | 6.40 | 6.24 | 6.16 | 5.33 |

**Supplementary Table 3. Percentage of EMG trials retained.**

| **Session** | **F** | **df (num)** | **df (denom)** | **p** | **n** |
| --- | --- | --- | --- | --- | --- |
| All- 10 trials | 1.28 | 9 | 250.03 | 0.25 | 15 |
| Session 1 | 1.77 | 3 | 83.43 | 0.16 | 15 |
| Session 2 | 0.50 | 3 | 97.01 | 0.68 | 15 |
| EEG Session | 0.87 | 1 | 42.00 | 0.36 | 15 |

**Supplementary Table 4. Results of mixed effects models on the percentage of retained background EMG trials during neurofeedback.**

| **Target (right) FDI** | | | | | |
| --- | --- | --- | --- | --- | --- |
|  | F | df(num) | df(denom) | p | n |
| Retention | 2.248 | 1 | 63.562 | 0.139 | 11 |
|  | | | | | |
| **OP** | | | | | |
|  | F | df(num) | df(denom) | p | n |
| Retention | 2.179 | 1 | 38.26 | 0.148 | 11 |
|  | | | | | |
| **ADM** | | | | | |
|  | F | df(num) | df(denom) | p | n |
| Retention | 0.173 | 1 | 37.809 | 0.68 | 11 |
|  | | | | | |
| **Left FDI** | | | | | |
|  | F | df(num) | df(denom) | p | n |
| Retention | 0.1 | 1 | 37.536 | 0.754 | 11 |

**Supplementary Table 5. F tests following mixed effects models comparing background EMG across conditions and blocks for the retention test conducted 6 months following initial training.**

| **Experimental Group** | | Strategies | |
| --- | --- | --- | --- |
| 1 | **Up**: I imagined grasping the fixation cross using a precision-grip, knowing that this will activate my index finger.  **Down**: I imagined kicking/pushing around a dead/ cut hand which was lying on a table | |  |
| 2 | **Down:** I tried to imaging my finger being cold and lifeless and impossible to move. I sometimes also imagined my finger not being a part of my hand.  **Up:** I imagined moving my finger in circles without really moving it and I was generally focused more on my hand and fingers. | |  |
| 3 | For the **Up** condition I had two strategies. One of it was imaging that I would play a piece of Piano (Beethoven‘s „1st Moonlight Sonata“ or Ludovico Einaudi’s „Fly“).  So I played the piano pieces in my mind focusing on the index finger part. The other one I used when I noticed that I became a bit tired, so then I started additionally to the piano thing to „talk“ mentally with my index to literally motivate it to move stronger and faster .  The **Down** condition was even less difficult. There it was enough to just make the finger and actually the whole hand feeling heavy. Letting it sink in into the cushion, feeling too heavy to be able to move anything and encapsulate it by the rest of the body. | |  |
| 4 | **Up:** Imagined movement of index finger. **Down:** Imagined Finger was paralyzed. | |  |
| 5 | Used the recommended strategies for **Up** and **Down** throughout as provided on participant information sheets. | |  |
| 6 | **Up:** Sometimes I imagined pushing my finger against an invisible wall. **Down:** I just tried to relax my whole hand as good as possible and that the hand is Kind of freezing and I could not move it. | |  |
| 7 | For the **Up** I was imagining holding my drumstick and repeatedly hitting the drums with high frequency which requires a lot of index finger control.  For the **Down** I was imagining having my whole hand & wrist cemented into a concrete block and having my whole limb immobilized in there. | |  |
| 8 | Used the recommended strategies for **Up** and **Down** throughout as provided on participant information sheets. | |  |
| 9 | During the **Up** condition I imagined being at a party and opening and closing my hands during dancing.  During the **Down** condition I tried to imagine being stuck in an Iceblock with my arm not being attached to my body but lying in front of me in another iceblock in a completely white clinical room with noise cancelling features. | |  |
| 10 | Used the recommended strategies for **Up** and **Down** throughout as provided on participant information sheets. | |  |
| 11 | **Up**: Imagination of pressing the thumb against the index finger. Focusing on the hand.  **Down:** Imagine that the hand is heavy and as it was unmovable. Trying to ‘forget’ the hand. | |  |
| 12 | Imagined a ball of energy in finger, and in **up** was getting bigger, in **down** getting smaller. | |  |
| 13 | **Up**: For the up condition, I imagined myself playing the cello, a rather easy tune, but for which I need the whole right hand.  **Down:** For this condition, I imagined my right hand to be really cold and numb. I imagine trying to warm it up with my left hand which had no effect. | |  |
| 14 | **Up:** For the up sessions, I have to concentrate on my finger. Concentrating on the arm does not work. Once again, I viewed the cross I immediately changed my attention to the right finger and think that it is active.  **Down:** In the down sessions, I have to imagine that my entire right arm is numb. When I concentrated on the finger specifically the result was not as constant. Once relaxed and viewing the cross I put my attention down my right arm, feeling the sensation that it is heavy and numb, almost detached from my body.  In either up or down session, if my attention waivers (even for a second) once the cross is present I often did not complete the task successfully. | |  |
| 15 | **Up:** Imagining pushing things with index finger or feeling things with pressure  **Down:** Ice water on finger, it really worked and finger felt really cold | |  |
| **Control Group** | | | |
| 1 | **Up:** Pushing a bell, rubbing my index finger on rough surfaces, doing arm wrestling with index fingers  **Down:** having my fingers inside bucket of ice, trying to reproduce a feeling of paralysis in my finger | |  |
| 2 | **Up:** Imagine an index finger is moving  **Down:** Imagine an index finger is in a bucket of cold water | |  |
| 3 | **Up:** imagined movement of finger left and right  **Down:** finger inside ice water frozen | |  |
| 4 | **Up:** Picking at something (e.g. tape)  **Down**: Sitting on my finger long enough so it falls asleep | |  |
| 5 | **Up:** Pressing the button on a video game controller  **Down:** Right index finger smashed in a door and paralyzed | |  |
| 6 | **Up:** Tapping of the finger on the board (tried to visualize my finger during the movement)  **Down:** My finger was dead or stuck in ice | |  |
| 7 | **Up:** Imagined forcefully moving my finger up and down  **Down:** Focused on the other fingers, giving no attention to the index finger to the extent I imagined it was not there | |  |
| 8 | **Up:** I imagined to either grab a root of a plant and pull it out of the ground or sometimes when this didn’t work anymore I imagined to pull me up on a rock with only my index finger.  **Down:** I tried to get the feeling as if my index finger was dead or fallen off by trying the blood to stop from getting there | |  |
| 9 | **Up:** Imagined changing between bending and extending the finger  **Down:** Paralyzed index finger | |  |
| 10 | **Up:** The finger goes up and down  **Down:** The finger isn’t there | |  |
| 11 | **Up:** I just imagined that my finger is moving up quickly and impulsively  **Down:** I imagined that I have no finger at all by visualizing myself with a cut finger. I imagined seeing the wound and touching it with other fingers | |  |
| 12 | **Up:** Writing numbers 8 and 0 in the air  **Down:** Paralyzed finger and trying to withdraw all the energy from it. | |  |
| 13 | **Up:** In this case I tried to think of my finger moving up and down while placed on the table. I was always thinking of that movement during the experiment.  **Down:** In this case I tried to be absorbed thinking totally different things apart from the experiment, duties, daily routine, friends etc, | |  |

**Supplementary Table 6: Participant Strategies.**

| **Neurofeedback Training/Testing Schedule** | | | | | | | | | | | | | | | | |
| --- | --- | --- | --- | --- | --- | --- | --- | --- | --- | --- | --- | --- | --- | --- | --- | --- |
| **Session 1** | | | | | | | | | | | | | | | | |
| Order  CB  across subjs | UP  *Day1* | RMT | Recruitment curve | BS | UP Block 1 | | Rest MEPs | UP Block 2 | | Rest MEPs | UP Block 3 | Rest MEPs | | UP Block 4 | | Rest MEPs |
|  | DOWN  *Day2* | RMT |  | BS | DN Block 1 | | Rest MEPs | DN Block 2 | | Rest MEPs | DN Block 3 | Rest MEPs | | DN Block 4 | | Rest MEPs |
|  | | | | | | | | | | | | | | | | |
| **Session 2** | | | | | | | | | | | | | | | | |
| Order  CB  across subjs | UP  *Day3* | RMT | BS | UP Block 5 | | Rest MEPs | | | UP Block 6 | Rest MEPs | UP Block 7 | Rest MEPs | UP Block 8 | | Rest MEPs | |
|  | DOWN  *Day4* | RMT | BS | DN Block 5 | | Rest MEPs | | | DN Block 6 | Rest MEPs | DN Block 7 | Rest MEPs | DN Block 8 | | Rest MEPs | |
|  | | | | | | | | | | | | | | | | |
| **Session 3 (EEG recording)** | | | | | | | | | | | | | | | | |
| Same day- 15 minute break between UP and DOWN measurements | UP  *Day5* | RMT | BS | UP Block 9 | | Rest MEPs | | | UP Block 10 | Rest MEPs |  | | | | | |
|  | DOWN  *Day5* | RMT | BS | DN Block 9 | | Rest MEPs | | | DN Block 10 | Rest MEPs |  |  |  |  |  |  |
|  | | | | | | | | | | | | | | | | |
| **Follow-up 6 months later** | | | | | | | | | | | | | | | | |
| **Session 4** | | | | | | | | | | | | | | | | |
| Order  CB  across subjs | UP  *Day6* | RMT | BS | Retention UP | | Rest MEPs 5 mins | | | Rest MEPs 10 mins | BS Right hemi | Opp hemi | Feedback free | | |  | |
|  | DOWN  *Day7* | RMT | BS | Retention DN | | Rest MEPs 5 mins | | | Rest MEPs 10 mins | BS Right hemi | Opp hemi | Feedback free | | |  |  |
|  | | | | | | | | | | | | | | | | |
| **Session 5** | | | | | | | | | | | | | | | | |
| Order  CB  across subjs | UP  *Day8* | RMT | SICI/LICI search | BS PP +SP | | Block 1 PP NF | | | Block 2 PP NF | Block 3 PP NF | |  | | | | |
|  | DOWN  *Day9* | RMT | SICI/LICI search | BS  PP +SP | | Block 1 PP NF | | | Block 2 PP NF | Block 3 PP NF | |  |  |  |  |  |

*Key: CB = Counterbalanced. BS = Baseline. NF= Neurofeedback. PP = Paired Pulse TMS. RMT= Resting Motor Threshold*

*Red line indicates beginning of testing for each session.*

*Grey shading indicates blocks in which MEP Neurofeedback was conducted.*

**Supplementary Table 7: Outline of experimental timeline for each day of testing.**
