# Supplementary material for: Neural activity related to volitional regulation of cortical excitability
Source: eLife. 2018 Nov 29;7:e40843. doi: 10.7554/eLife.40843 (PMC6294548; doi:10.7554/eLife.40843)
Supplement: Transparent reporting form [file elife-40843-transrepform.docx]

***eLife’s* transparent reporting form**

We encourage authors to provide detailed information *within their submission* to facilitate the interpretation and replication of experiments. Authors can upload supporting documentation to indicate the use of appropriate reporting guidelines for health-related research (see [EQUATOR Network](http://www.equator-network.org/%20)), life science research (see the [BioSharing Information Resource](https://biosharing.org/)), or the [ARRIVE guidelines](http://www.plosbiology.org/article/info:doi/10.1371/journal.pbio.1000412) for reporting work involving animal research. Where applicable, authors should refer to any relevant reporting standards documents in this form.

If you have any questions, please consult our Journal Policies and/or contact us: [editorial@elifesciences.org](mailto:editorial@elifesciences.org).

**Sample-size estimation**

- You should state whether an appropriate sample size was computed when the study was being designed
- You should state the statistical method of sample size computation and any required assumptions
- If no explicit power analysis was used, you should describe how you decided what sample (replicate) size (number) to use

Please outline where this information can be found within the submission (e.g., sections or figure legends), or explain why this information doesn’t apply to your submission:

Sample size was estimated prior to study design, and this analysis is reported in the supplementary material. This is the caption below the relevant figure: “**Figure1-figure supplement 1: Power analysis to compute sample size.** Conducted using G*Power software version 3.1, based on the inclusion of 2 groups, an alpha value of 0.05, and an effect size of 0.81 (Cohen’s d reported by Majid et al 2017 in a study training voluntary modulation of MEP amplitude, for an F-test comparing effect of whether the MEP was cued or uncued). As our core findings are communicated using F-tests following mixed effects models, we selected to perform this power analysis based on the F-tests family.”

This resulted in a recommended total sample size of 27. In total we tested 28 participants.

The power analysis is referred to in the main text at line 517-518

**Replicates**

- You should report how often each experiment was performed
- You should include a definition of biological versus technical replication
- The data obtained should be provided and sufficient information should be provided to indicate the number of independent biological and/or technical replicates
- If you encountered any outliers, you should describe how these were handled
- Criteria for exclusion/inclusion of data should be clearly stated
- High-throughput sequence data should be uploaded before submission, with a private link for reviewers provided (these are available from both GEO and ArrayExpress)

Please outline where this information can be found within the submission (e.g., sections or figure legends), or explain why this information doesn’t apply to your submission:

There are no replications included in the current set of experiments.

An outlier was identified in the EEG data on the basis of excessive gamma activity, exceeding 3x standard deviations greater than the rest of the dataset. This participant (code NF7) was excluded from subsequent EEG analyses. This is described in the text at lines 273-274.

**Statistical reporting**

- Statistical analysis methods should be described and justified
- Raw data should be presented in figures whenever informative to do so (typically when N per group is less than 10)
- For each experiment, you should identify the statistical tests used, exact values of N, definitions of center, methods of multiple test correction, and dispersion and precision measures (e.g., mean, median, SD, SEM, confidence intervals; and, for the major substantive results, a measure of effect size (e.g., Pearson's r, Cohen's d)
- Report exact p-values wherever possible alongside the summary statistics and 95% confidence intervals. These should be reported for all key questions and not only when the p-value is less than 0.05.

Please outline where this information can be found within the submission (e.g., sections or figure legends), or explain why this information doesn’t apply to your submission:

Alongside all summary statistics and inferential tests in the results section, exact p values are reported for all tests. In cases where several tests were conducted and all were non-significant, the exact p value of the smallest result was reported, with the indication that all others were larger than this (eg. all<0.15). In cases where multiple comparisons issues arose, we conducted False Discovery Rate (FDR) corrections. Where conducted, these are indicated in the text by ‘p_FDR_=’. The ‘n’ value for all tests are reported, and the specific test used was always indicated nearby in the supporting text. Dispersion figures (Standard deviations) are reported where appropriate, and in figures all are shown with error bars for Standard error of the mean. Appropriate measures of effect size (Cohen’s d) are reported for all key findings. Raw data from individual subjects are shown in the Figure Supplements (Figure 2-figure supplements 2,3,4, Figure 5-figure supplements 3 and 4) but all other figures show means across all subjects.

(For large datasets, or papers with a very large number of statistical tests, you may upload a single table file with tests, Ns, etc., with reference to sections in the manuscript.)

**Group allocation**

- Indicate how samples were allocated into experimental groups (in the case of clinical studies, please specify allocation to treatment method); if randomization was used, please also state if restricted randomization was applied
- Indicate if masking was used during group allocation, data collection and/or data analysis

Please outline where this information can be found within the submission (e.g., sections or figure legends), or explain why this information doesn’t apply to your submission:

Participants were allocated randomly to either the experimental (n=15) or control (n=13) group, and were blinded concerning the group they were in during testing, until debriefing.

**Additional data files (“source data”)**

- We encourage you to upload relevant additional data files, such as numerical data that are represented as a graph in a figure, or as a summary table
- Where provided, these should be in the most useful format, and they can be uploaded as “Source data” files linked to a main figure or table
- Include model definition files including the full list of parameters used
- Include code used for data analysis (e.g., R, MatLab)
- Avoid stating that data files are “available upon request”

Please indicate the figures or tables for which source data files have been provided:

Source data for all figures and analyses reported in the text are provided, hosted on ETH Zurich’s University Library data server with the Digital object identifier: <https://doi.org/10.3929/ethz-b-000300799>

Relevant analysis (and in some cases plotting) scripts are also provided alongside the data.
